# Supplementary material for: A guide to selecting high-performing antibodies for ARID2 (UniProt ID: Q68CP9) for use in western blot, immunoprecipitation, and immunofluorescence
Source: F1000Res. 2026 Apr 24;15:614. [Version 1] doi: 10.12688/f1000research.179367.1 (PMC13224947; doi:10.12688/f1000research.179367.1)
Supplement: Peer review history

# Reviewer report: Reviewer response for version 1

**DOI:** 10.5256/f1000research.197872.r480017

**Published:** 2026-05-29

**Authors:** Deborah Moshinsky

**Affiliations:** 1 Institute for Protein Innovation, Boston, Massachusetts, USA

ARID2 contributes to the regulation of gene expression through interactions with DNA and nucleosomes. 6 ARID2 commercial antibodies were characterized by Western Blot, IP, and IF using standardized protocols against cells and lysates with wild type antigen in addition to those with the antigen knocked out.  The results were clearly shown for the readers' interpretation.  The authors purposely did not engage in result interpretation, but rather left the results for the reader to use in addition to guidance on result interpretation.  These data are useful in determining which commercial antibodies are active under the conditions tested.

Are sufficient details of methods and materials provided to allow replication by others?

Yes

Is the rationale for creating the dataset(s) clearly described?

Yes

Are the datasets clearly presented in a useable and accessible format?

Yes

Are the protocols appropriate and is the work technically sound?

Yes

Reviewer Expertise:

Antibody Characterization and Validation

I confirm that I have read this submission and believe that I have an appropriate level of expertise to confirm that it is of an acceptable scientific standard.

# Reviewer report: Reviewer response for version 1

**DOI:** 10.5256/f1000research.197872.r479994

**Published:** 2026-05-13

**Authors:** Karen Bowman

**Affiliations:** 1 University of Leicester, Leicester, England, UK

The study involved characterisation of six commercial antibodies, from four different companies, for the AT-rich interactive domain-containing protein 2 (ARID2) protein for use in western blot, immunoprecipitation, and immunofluorescence. The study formed part of a wider YCharOS collaboration to characterise commercial antibodies, which are renewable (recombinant and monoclonal), for human proteins using standardised protocols with the intention of improving antibody reproducibility issues. As a Data Note, it allows the direct comparison of the performance of commercially available antibodies to the ARID2 protein to aid scientists in choosing the most appropriate antibody for their studies. For example, in understanding how disease-related mutations affect the protein’s biochemical and cellular characteristics. This comparison would be of particular use in the study of chromatin architecture and genomic stability, as well as the study of cancer and neurodevelopmental disorders.

 A committee of industry and academic representatives have endorsed the platform used, and the protocols are consistent with those in general use. The platform consisted of identification of a human cell line suitable for antibody characterisation studies i.e., with adequate levels of the ARID2 protein to generate a measurable signal. Using the DepMap (Cancer Dependency Map Portal, RRID: SCR_017655) transcriptomics database, they found that the commercially available HAP1 WT cell line expresses *ARID2* transcript at a level above the average range for cancer cells analysed, and therefore, was a logical choice for their study. Knockdown of the target gene in HAP1 cells was used to provide an appropriate negative control HAP1 cell line ( *ARID2* KO). The final step was a series of antibody characterisation procedures, limited to the most common research uses of antibodies. The interpretation of the results is left to the reader, with the study providing unbiased guidance. However, it gave some indication of the optimum dilutions of commercial antibodies. Limitations of the study are clearly stated.

 It would be useful to know if any of the antibodies bound to mutant versions of ARID2. Since only six antibodies were studied, perhaps a comparison of the antibodies with other cell lines with mutations in *ARID2* could have been added to determine effects on antibody-epitope binding.

Are sufficient details of methods and materials provided to allow replication by others?

Yes

Is the rationale for creating the dataset(s) clearly described?

Yes

Are the datasets clearly presented in a useable and accessible format?

Yes

Are the protocols appropriate and is the work technically sound?

Yes

Reviewer Expertise:

Anti-cancer drug discovery and development. Diagnostics.

I confirm that I have read this submission and believe that I have an appropriate level of expertise to confirm that it is of an acceptable scientific standard.
